# Characterization of the bovine salivary gland transcriptome associated with *Mycobacterium avium* subsp. *paratuberculosis* experimental challenge

**DOI:** 10.1186/s12864-019-5845-4

**Published:** 2019-06-13

**Authors:** Sanjay Mallikarjunappa, Mounir Adnane, Paul Cormican, Niel A. Karrow, Kieran G. Meade

**Affiliations:** 1 Animal & Bioscience Research Department, Animal & Grassland Research and Innovation Centre, Teagasc, Grange, Co. Meath, Ireland; 2 Department of Animal Biosciences, Centre for Genetic Improvement of Livestock, University of Guelph, Guelph, Ontario N1G 2W1 Canada; 3 Institute of Veterinary Sciences, Ibn Khaldoun University, Tiaret, Algeria

**Keywords:** Johne’s disease, Cattle, Salivary glands, RNA-Seq, Saliva, Biomarkers

## Abstract

**Background:**

*Mycobacterium avium* subsp. *paratuberculosis* (MAP), the etiologic agent of Johne’s disease is spread between cattle via the fecal-oral route, yet the functional changes in the salivary gland associated with infection remain uncharacterized. In this study, we hypothesized that experimental challenge with MAP would induce stable changes in gene expression patterns in the salivary gland that may shed light on the mucosal immune response as well as the regional variation in immune capacity of this extensive gland. Holstein-Friesian cattle were euthanized 33 months’ post oral challenge with MAP strain *CIT003* and both the parotid and mandibular salivary glands were collected from healthy control (*n* = 5) and MAP exposed cattle (*n* = 5) for histopathological and transcriptomic analysis.

**Results:**

A total of 205, 21, 61, and 135 genes were significantly differentially expressed between control and MAP exposed cattle in dorsal mandibular (M1), ventral mandibular (M2), dorsal parotid (P1) and ventral parotid salivary glands (P2), respectively. Expression profiles varied between the structurally divergent parotid and mandibular gland sections which was also reflected in the enriched biological pathways identified. Changes in gene expression associated with MAP exposure were detected with significantly elevated expression of *BoLA DR-ALPHA*, *BOLA-DRB3* and complement factors in MAP exposed cattle. In contrast, reduced expression of genes such as polymeric immunoglobin receptor (*PIGR*), *TNFSF13*, and the antimicrobial genes lactoferrin (*LF*) and lactoperoxidase (*LPO*) was detected in MAP exposed animals.

**Conclusions:**

This first analysis of the transcriptomic profile of salivary glands in cattle adds an important layer to our understanding of salivary gland immune function. Transcriptomic changes associated with MAP exposure have been identified including reduced LF and LPO. These critical antimicrobial and immunoregulatory proteins are known to be secreted into saliva and their downregulation may contribute to disease susceptibility. Future work will focus on the validation of their expression levels in saliva from additional cattle of known infection status as a potential strategy to augment disease diagnosis.

**Electronic supplementary material:**

The online version of this article (10.1186/s12864-019-5845-4) contains supplementary material, which is available to authorized users.

## Background

*Mycobacterium avium* subsp. *paratuberculosis* (MAP) is the etiological agent of Johne’s disease (JD) in cattle. JD is chronic in nature and manifests as granulomatous enteritis in MAP-infected animals. The fecal-oral route is the primary mode of MAP transmission and calves less than 6 months of age are known to be highly susceptible to MAP infection [1, 2] The pathogenesis of JD involves a long latent subclinical phase and a symptomatic clinical phase. Although asymptomatic, shedding of MAP occurs intermittently during the sub-clinical phase causing disease dissemination. During the clinical phase, infected animals present with profuse watery diarrhea, loss of weight and a significant reduction in milk production, eventually causing wasting and death [3].

JD is prevalent worldwide and causes severe economic losses to the dairy industry due to associated production losses and animal welfare concerns [4]. Although whether MAP can cause Crohn’s disease is controversial and debatable, isolation of MAP from the intestines of patients suffering from Crohn’s disease has also raised public health concerns [5].

Numerous factors contribute to poor control of JD including a poor understanding of factors influencing host susceptibility, diagnostics with limited sensitivity, and the absence of an efficacious vaccine that can clear MAP infection [6]. Current JD control measures include culling MAP positive animals and improving management practices aimed at reducing the risk of contamination within and across herds. Fecal culture, milk and serum ELISA, fecal PCR, and IFN-γ assay are the commonly employed diagnostic tests, often used in conjunction, to diagnose JD. Milk and serum ELISA detect the presence of MAP-specific antibodies and are the most commonly used JD diagnostic method in field conditions because of the quick turnaround time, but their sensitivity is low [7], particularly during the subclinical stage of infection when antibody response is low in the infected animals. Fecal culture has a very high specificity of 99% but requires a long incubation period of 8–16 weeks before an animal can be diagnosed as positive or negative for JD and also lacks sensitivity (~ 60%) during the subclinical stages when shedding is intermittent [8]. Fecal PCR that detects MAP-specific DNA is slightly more sensitive than fecal culture and has similar specificity [9] but it does not confirm the presence of viable MAP organisms. The IFN-γ assay involves measuring IFN-γ that drives the cell-mediated immune response in the infected animal [10]; IFN-γ is released from the lymphocytes after *ex-vitro* challenge with MAP antigen and is measured by ELISA. IFN-γ assay has the potential to detect early phase of MAP exposure; however, the results are highly variable [11] .

Given the difficulties associated with the currently available JD diagnostic techniques, there is a continued need to explore new diagnostic approaches. One such new approach would be the identification of salivary biomarkers that can distinguish MAP exposed versus non-exposed cattle. Cattle produce over 220 L of saliva per day [12]; saliva could hold promise for the routine and accessible profiling of diagnostic biomarkers [13]. In addition, salivary secretions could have enormous significance for immuno-protection of the oral cavity, as well as the regulation of the intestinal microflora [14, 15]. However, detail in cattle in this regard is scant, and very little information is available regarding the functional competence of this complex and extensive gland.

Previous studies in humans and mice have revealed expression of antimicrobial peptides such as defensins and cathelicidins in parotid, mandibular and sublingual salivary glands and their subsequent secretion in saliva [16–18]. A study by Ang et al. [19] has given insights into the complexity of the secreted proteins in bovine saliva, via the identification of 402 proteins. However, disease-associated changes have not been previously explored in cattle. In pigs, the expression of the acute phase protein C-reactive protein (CRP) in saliva has been used to discriminate healthy pigs from those with experimentally-induced inflammation [20]. All these findings illustrate the informative value of biomolecules in saliva associated with health and disease, and hint at the potential utility of such molecules for improving disease diagnosis [13, 21, 22].

Our study was based on the hypothesis that profiling the salivary gland transcriptome between control cattle and matched but MAP exposed cattle may identify stably differentially expressed genes, which if secreted in saliva, could signpost potential oral salivary biomarkers for early detection of MAP exposure and improved JD diagnosis.

## Results

### Sequencing and alignment of reads to bovine reference genome

A total of 39 salivary gland samples, representing two regions of both the parotid - dorsal parotid (P1) and ventral parotid (P2) regions and the mandibular - dorsal mandibular (M1) and ventral mandibular (M2) salivary glands from control and MAP exposed cattle were used for RNA-seq. An average of 114 million paired end reads (average ± SD = 114,426,881 ± 8,388,320 were generated. Post mapping, the number of reads that uniquely mapped to the *Bos taurus* reference genome (BTA_UMD3.1) in each sample was greater than 90%. Reads that were mapped to multiple regions were excluded from downstream differential gene expression analysis. Mapping statistics for each sample are provided in Additional file 1: Table S1.

### Principal component analysis (PCA)

**Fig. 1:**
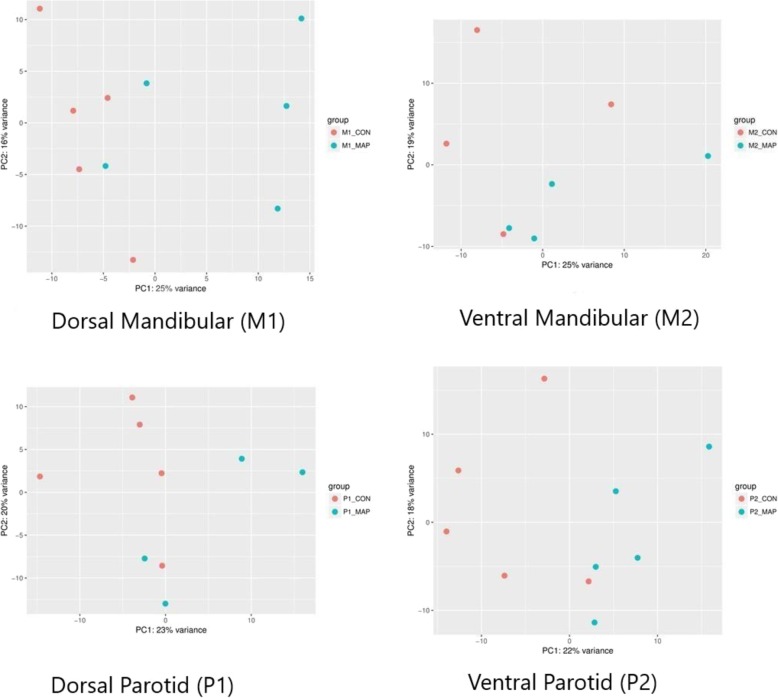
Principal Component Analysis (PCA) plot of the DEG dataset in Dorsal mandibular salivary gland extremity (M1); Ventral mandibular salivary gland extremity (M2); Dorsal parotid salivary gland extremity (P1) and Ventral parotid salivary gland extremity (P2) from control and MAP exposed cattle. The control (red) and MAP exposed (blue) samples are plotted along the first two principal component axes (PC1 and PC2)

PCA of normalized read counts was performed to compare sample clustering between control and MAP exposed samples within each salivary gland group and to identify outliers. Based on PCA, two M2 salivary gland samples (sample #2402, #2176) and one from the P1 group (sample #2420) that did not cluster within their respective groups were deemed outliers and were excluded from downstream differential gene expression analysis. Figure 1 depicts the PCA plots that show the samples clustering by control vs. MAP exposed group in all the 4 salivary gland groups.

### Histopathology

**Fig. 2:**
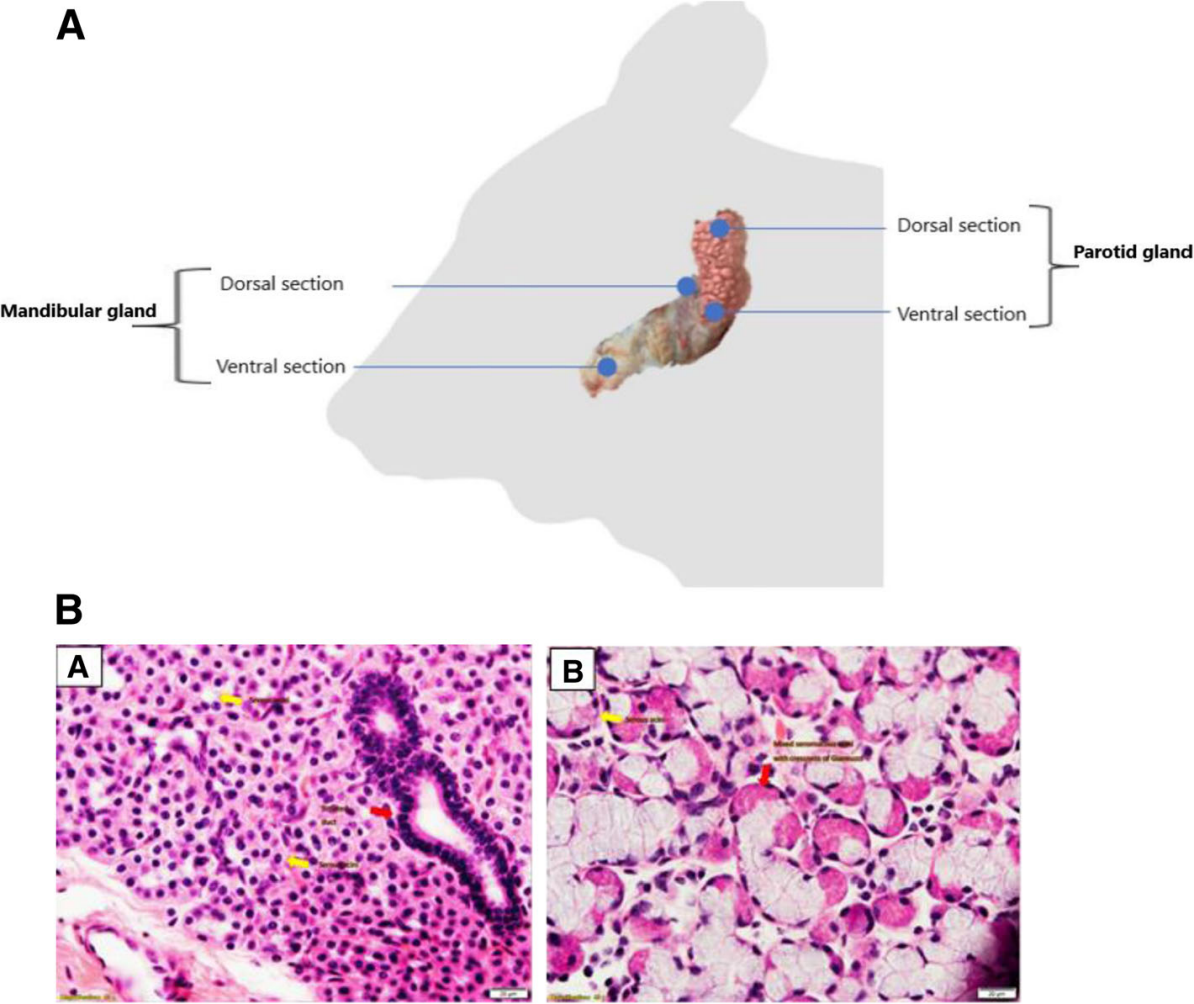
**a** Salivary glands sampling. After euthanasia, the head was positioned upside down and the skin between jaws was incised using sterile disposable scalpel. Then, diagonal incision was made from the ear to join the first incision and the skin was removed from one side to expose the adjacent tissues. Fatty tissue was incised at the site of targeted salivary glands. Parotid and mandibular glands were located at one side and two samples were collected at dorsal and ventral anatomical sections from each gland. **b**: **a**: Parotid gland; Pure serous acini consisting of rectangular granular cells with central nuclei. Central lumen hardly visible (yellow arrow). Striated duct with columnar cells with central nuclei and basal-striated appearance (red arrow). **b** Mandibular gland; Pure serous acini consisting of triangular granular cells with their base directed outwards and basal nuclei (yellow arrow). Mixed seromucous acini with crescents of Giannuzzi (red arrow). Bar length 20 um

No histopathological changes related to MAP infection were observed in salivary glands under H&E staining. Similarly, Ziehl-Neelsen (ZN) staining did not identify acid-fast MAP in any of the salivary gland samples. Histopathological image of two representative samples from parotid and mandibular salivary gland are shown in Fig. 2b. The structural difference between the two glands was evident with parotid gland comprising of pure serous acini consisting of rectangular granular cells with central nuclei and a hardly visible central lumen. Whereas, the mandibular gland comprised of pure serous acini consisting of triangular granular cells with their base directed outwards and with basal nuclei. Mixed seromucous acini with crescents of Giannuzzi were also seen in mandibular glands. The observed structural differences between the two major salivary glands is reflective of their functional and secretory adaptations.

### Differential gene expression analysis

**Fig. 3:**
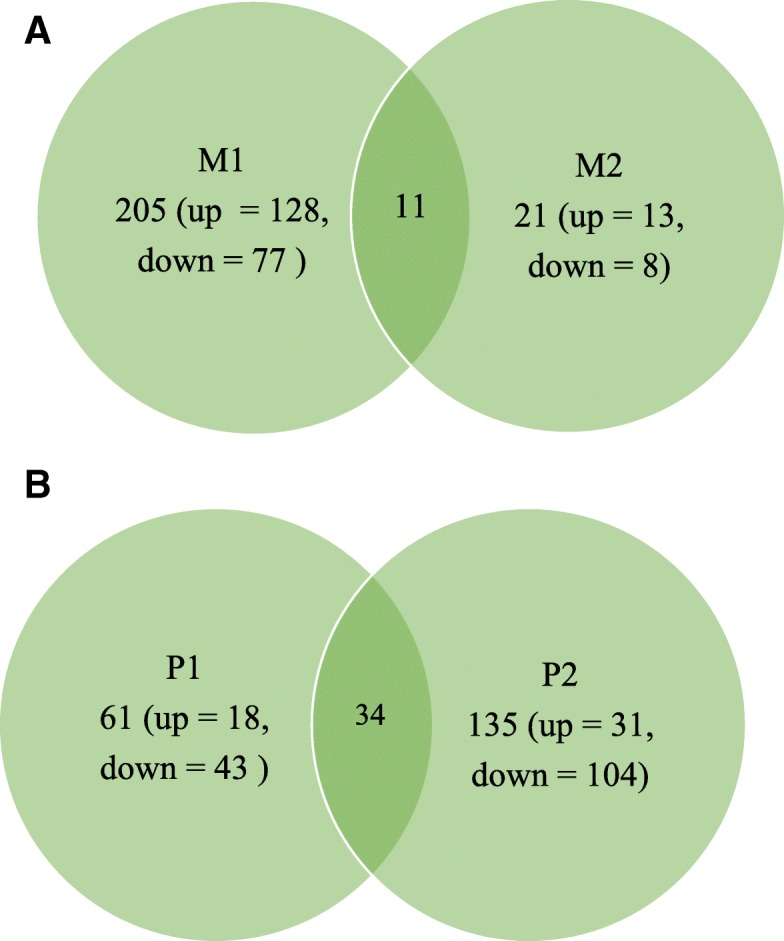
**a** Venn diagram comparing the number of DEGs identified in M1 and M2 salivary gland regions along with the intersection indicating the number of common DEGs. up = upregulated or down = downregulated in corresponding salivary gland group. **b** Venn diagram comparing the number of DEGs identified in P1 and P2 salivary gland along with the intersection indicating the number of common DEGs. up = upregulated or down = downregulated in corresponding salivary gland group

**Fig. 4:**
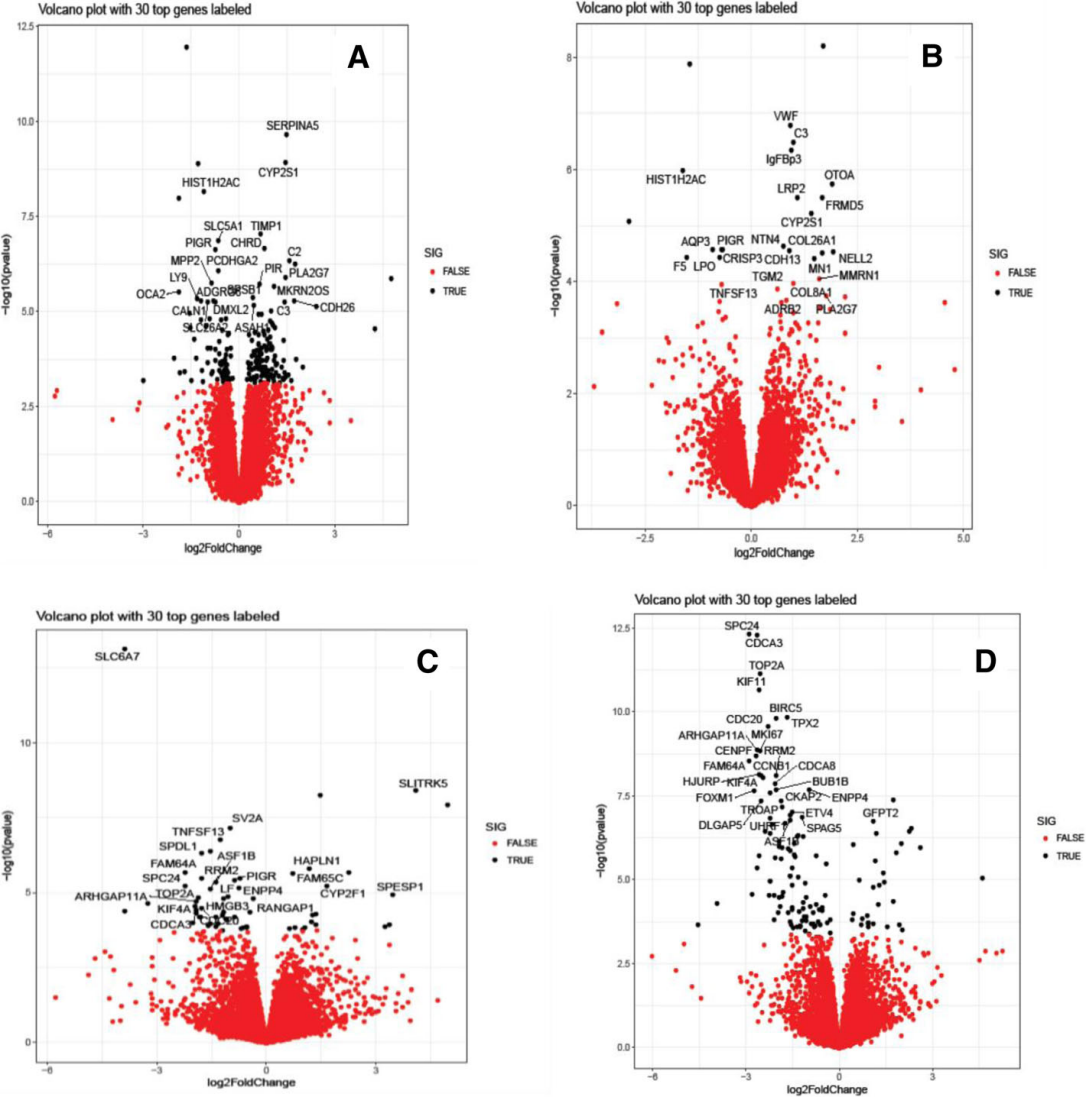
Volcano plot of differential expression (−log10 *p*-value vs log2fold change) in dorsal mandibular salivary gland (M1) (**a**), ventral mandibular salivary gland extremity (M2) (**b**), dorsal parotid salivary gland (P1) (**c**) and ventral parotid salivary gland extremity (P2) (**d**), respectively. Genes with an FDR < 0.05 are highlighted in black with top 30 of them labeled by their names

Differentially expressed genes (DEGs) between control and MAP exposed cattle in mandibular and parotid salivary glands were determined using DeSeq2 software. A False Discovery Rate (FDR) of 5% was used to correct for multiple testing. The identified DEGs were found to be significant with a *p*_*adj*_ *< 0.05*. In the M1 salivary gland group, a total of 205 genes were differentially expressed between the two groups, of which the expression of 128 genes was upregulated and 77 genes were downregulated in the MAP exposed animals. In M2 group, 21 genes were differentially expressed with 13 genes being upregulated and 8 genes with a downregulated expression in MAP exposed animals. A total of 11 DEGs were found to be common between M1 and M2 groups with their log2 fold-change expression observed in the same direction (Fig. 3a). Figure 4 (a and b) depict the volcano plot indicating the log2 fold-change of the top 30 differentially expressed genes in M1 and M2 salivary glands, respectively. Overall, in both M1 and M2 salivary gland groups, majority of the identified DEGs had their expression upregulated in MAP exposed animals.

The number of DEGs identified in P1 and P2 groups was 61 and 135, respectively. Within P1 group, a total of 18 and 43 genes were up- and down-regulated, respectively, in MAP exposed animals; whereas, in P2 group, 31 and 104 genes were upregulated and downregulated, respectively. The number of DEGs that were common between P1 and P2 groups was found to be 34 with their log2 fold-change expression observed in the same direction (Fig. 3b). Figure 4 (c and d) depict the volcano plot indicating the log2 fold-change of the top 30 differentially expressed genes in P1 and P2 salivary glands, respectively. Contrary to mandibular salivary glands, the expression of the majority of the identified DEGs was downregulated in MAP exposed animals in both P1 and P2 salivary gland groups. Additional file 2: Table S2 provides the summary of the identified DEGs in all the 4 salivary gland groups.

**Fig. 5:**
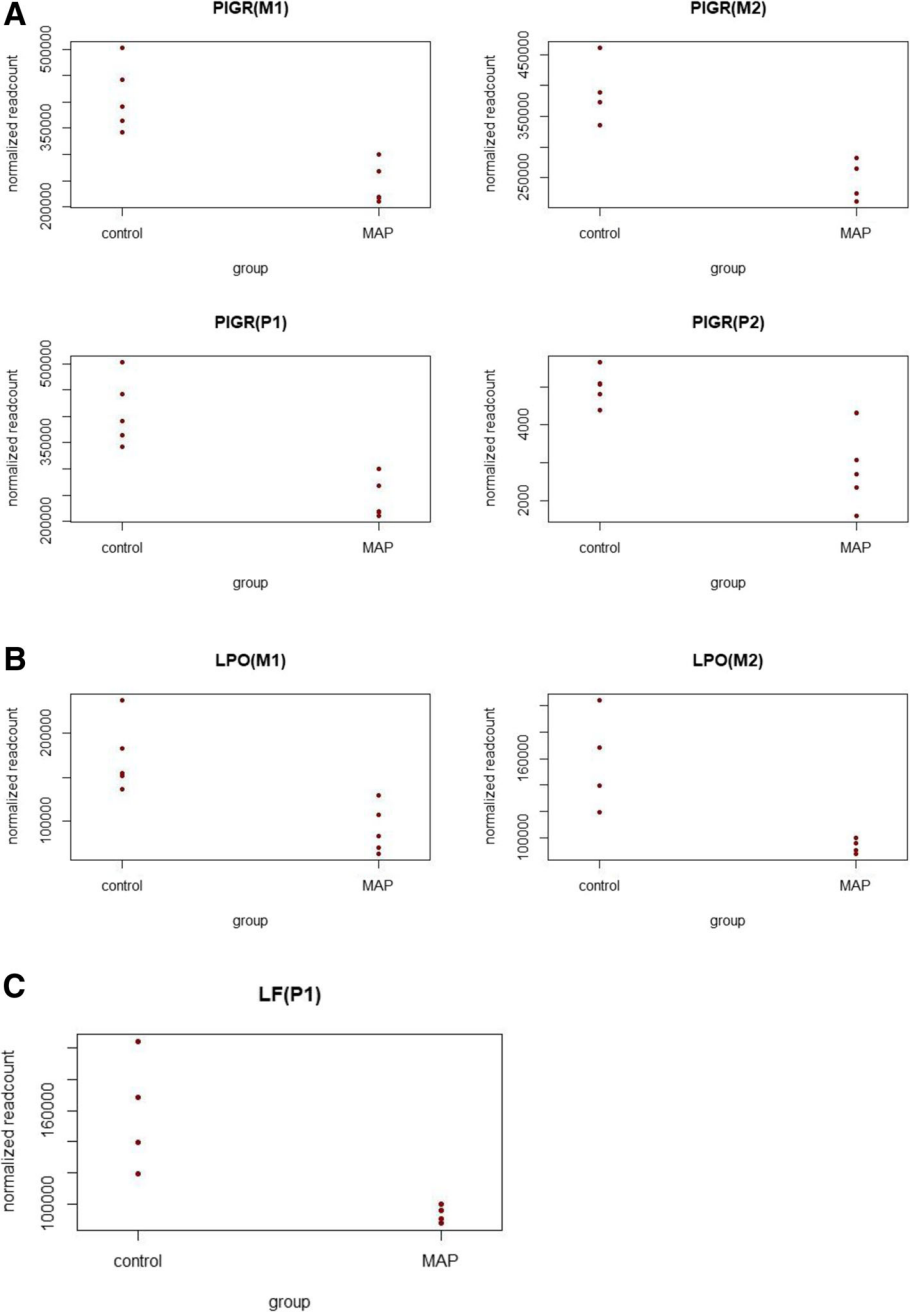
**a** Expression of *Polymeric Immunoglobulin Receptor* (PIGR) in salivary glands (salivary gland group in the paranthesis). The expression was downregulated in MAP infected animals in all the salivary gland groups; **b** Expression of *lactoperoxidase* (LPO) in M1 and M2 salivary gland groups (salivary gland group in the paranthesis). LPO expression was downregulated in MAP-infected animals in M1 and M2 salivary gland groups; **c** Expression of *lactoferrin* (LF) in P1 salivary gland group (salivary gland group in the paranthesis). LF expression was downregulated in MAP-infected animals in P1 salivary gland group

Polymeric immumoglobin receptor (*PIGR*) gene was significantly differentially expressed in all the 4 salivary gland groups with its expression downregulated in MAP exposed animals (Fig. 5a). Figure 5b and c illustrate the expression of highly abundant and differentially expressed antimicrobial genes lactoperoxidase (in M1 and M2) and lactoferrin (in P1 group) respectively.

### Gene ontological analysis of DEGs

**Fig. 6:**
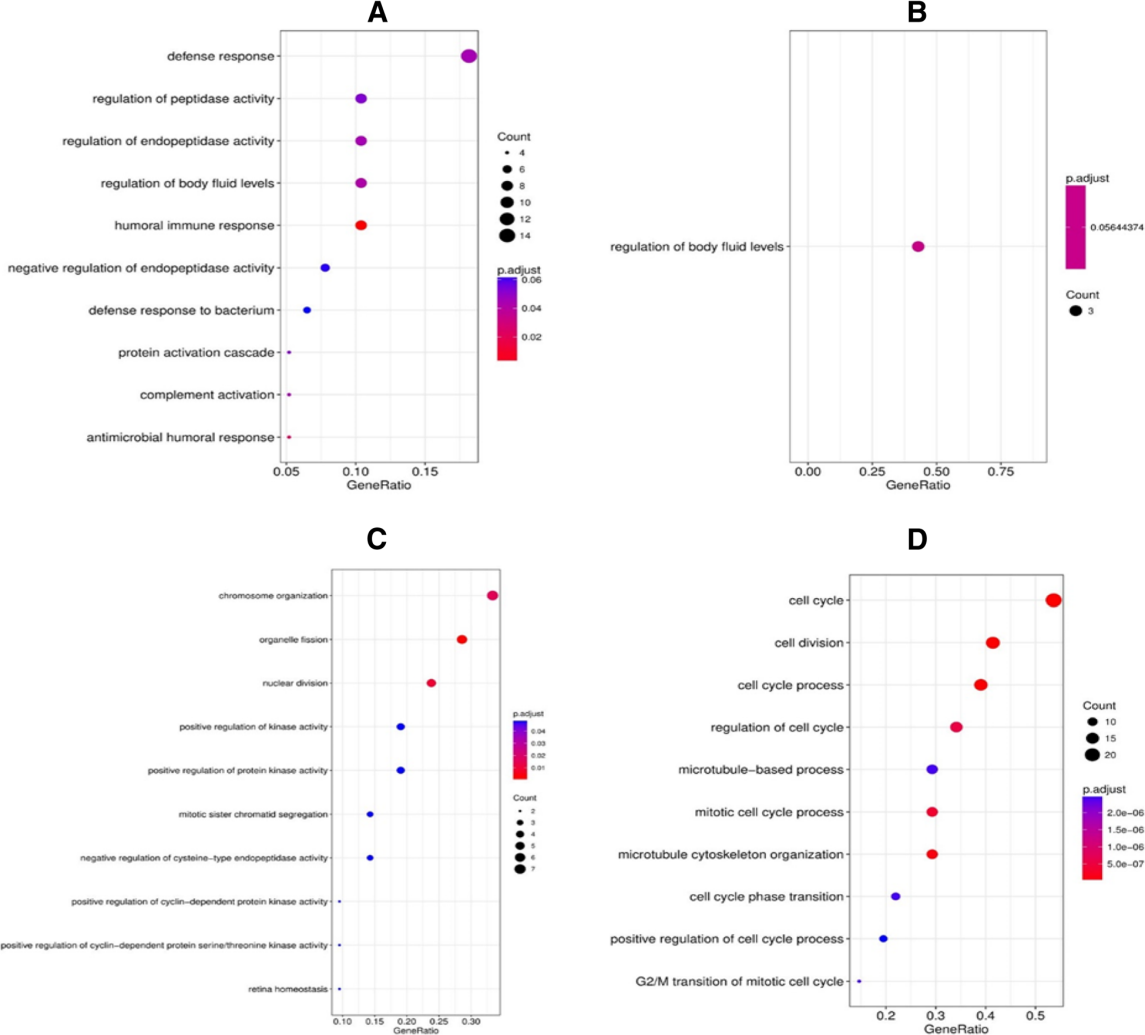
Biological processes enriched among DEGs in dorsal mandibular salivary gland extremity (M1) (**a**), ventral mandibular salivary gland extremity (M2) (**b**), dorsal parotid salivary gland extremity (P1) (**c**) and ventral parotid salivary gland extremity (P2**)** (**d**)

Gene ontology (GO) analysis identified the different functional groups enriched among DEGs in each salivary gland group. Four molecular functions and 12 biological processes were enriched among the DEGs in M1 group; while in M2 group, one biological process and one cellular component were over represented among the identified DEGs. Fifty-four biological processes, 18 cellular components and 23 molecular functions were enriched among DEGs in P1 group; whereas in P2 salivary gland group, a total of 84 biological processes, 38 cellular components and 7 molecular functions were enriched among DEGs. Figure 6 illustrates the biological pathways enriched among DEGs within each salivary gland group.

### KEGG pathway analysis

KEGG pathway analysis identified over-representation of 10 pathways in M1 salivary glands. In both M2 and P1 salivary glands, the ‘complement and coagulation cascades’ (KEGG ID = bta04610) was enriched. Five pathways were over-represented in P2 salivary gland. Structural divergence observed under histopathology between parotid and mandibular salivary glands was also reflected in the biological processes enriched among the DEGs within each salivary gland (Fig. 6). While DEGs in parotid salivary gland influenced processes such as cell division and cell cycle regulation for example, mandibular salivary gland DEGs were immunologically active in terms of enrichment of immune biological processes such as defense response, humoral immune response, defense response to bacterium and complement activation. All the identified KEGG pathways are listed in Additional file 3: Table S3.

## Discussion

Despite the spread of multiple infectious agents via the fecal-oral route, the functional and specifically the immune capacity of mucosal tissues within the oral cavity remains poorly understood, particularly in livestock species. Johne’s disease, caused by *Mycobacterium avium* subsp. *paratuberculosis* is spread via shedding of mycobacteria onto infected pasture, where ingestion sustains the cycle of infection. MAP has been previously detected in bovine saliva [23], but yet the immune capacity and changes associated with disease in the salivary gland have not previously been characterised. Therefore, in this study, we hypothesised that experimental infection with MAP would lead to persistent immune system changes that could be detected, initially, at salivary gland transcriptomic level in MAP exposed cattle. Such analysis would be very informative from a functional point of view but changes could also form the basis of improved disease surveillance and diagnostic approaches.

Cattle produce over 220 L of saliva per day [12], and it is plausible that secretions from the parotid and mandibular glands are likely to play a role in early innate resistance to infection as well as in immunoprotection of the oral cavity and digestive tract [15]. Whereas, extensive analysis of the digestive tract has shed light on the immune mechanisms by which the host immune system recognizes and responds to MAP infection [24], our knowledge of the immune capacity of the salivary glands remains rudimentary by comparison.

Both parotid and mandibular regions of the salivary gland differ in both structure and function. The parotid gland is of ectodermal origin, whereas the mandibular gland is of endodermal derivation and is relatively larger than the parotid gland [25]. While the glandular acini of the parotid gland are purely serous, the mandibular gland has a mixture of serous and mucus-secreted acini [25, 26]. Unlike parotid glands, mandibular glands produce large amount of mucus that contains high levels of immune molecules such as lactoferrin, cystatins and immune-active mucins [22]. Histopathological analysis illustrated the structural divergence of the major salivary glands with parotid gland serous acini consisting of rectangular granular cells with central nuclei. In mandibular gland, pure serous acini consisted of triangular granular cells with basal nuclei and also mixed seromucous acini with crescents of Giannuzzi. The structural changes between the two salivary gland types were also reflected at a transcriptomic level. Whereas no large differences were observed in the functional capacity of 4 salivary gland groups in terms of expression of number of gene transcripts both between and within salivary gland groups, significantly different numbers of genes were identified as DEG between the parotid and mandibular salivary gland sections. While the number of DEGs identified in P1 and P2 salivary gland groups was 61 and 35 respectively, the number was higher in mandibular salivary gland with a total of 205 and 128 genes identified as differentially expressed in M1 and M2 salivary gland sections, respectively. Differences in the number of common DEGs shared within salivary gland sections was also observed. A total of 34 DEGs were common between P1 and P2 salivary gland sections. The common genes identified included genes such as *PIGR* and *TNFSF13.* Eleven DEGs were found to be common between M1 and M2 sections. This list comprised of some key immunoregulatory genes such as *PIGR*, *C3*, and antimicrobial *LPO*.

### MAP exposure associated changes in salivary gland gene expression

Within each gland, although small numbers of genes were differentially expressed, a number of DEGs with important immunological properties were identified. Two genes - *PIGR* (Polymeric Immunoglobulin Receptor) and ENSBTAG00000026758 were differentially expressed in all four salivary gland sections. While ENSBTAG00000026758 is uncharacterized, *PIGR* function is well documented; *PIGR* plays an important role in mucosal immunity as it mediates the transfer of secretory IgA antibodies across intestinal epithelial cells to mucosal surfaces where IgA antibodies serve as first line of defense against microbes [27]. In this study, *PIGR* expression was downregulated in MAP exposed animals and this was consistent in all the salivary gland sections. Although there is no evidence in literature to support negative regulation of *PIGR* expression by MAP, it would be interesting to know if MAP favors this to promote its uptake by the host cell, particularly at the level of the intestinal mucosa where MAP is phagocytosed. To support this statement, *PIGR* was one of the genes identified in KEGG pathway ‘intestinal immune network for IgA production’ (KEGG ID = bta04672;). It has also been reported that salivary IgA is a proxy indicator of intestinal immune induction [28]. It can therefore be speculated that *PIGR* downregulation decreases IgA secretion at mucosal surfaces. Furthermore, the secretion of *PIGR* in bovine saliva has been reported [19] and there is a need to further investigate the role of *PIGR* as a potential salivary biomarker to identify MAP exposed cattle.

Expression of another gene, *TNFSF13*, was downregulated in M1, P1 and P2 salivary glands of MAP exposed animals; *TNFSF13*, also known as APRIL, is a proliferation-inducing ligand and is a member of BAFF system molecules that plays a vital role in mature B-cell survival and in secretion of IgA antibody [29]. Similar to *PIGR*, *TNFSF13* expression was downregulated in salivary glands and was also identified in KEGG pathway ‘intestinal immune network for IgA production’. While this indicates the role of both *TNFSF13* and *PIGR* in conferring mucosal immunity via secretion of IgA and its transfer, their downregulation in MAP-infected animals could therefore be speculated as a mechanism employed by MAP to evade mucosal immunity and to promote its survival. The other two DEGs identified in this pathway were two MHC genes *BoLA DR-ALPHA* and *BOLA-DRB3,* with their expression being upregulated in the M1 salivary gland of MAP exposed cattle. These MHC genes were also identified in another KEGG pathway ‘phagosome’ (KEGGID = bta04145).

### Significant reduction in gene expression of the highly abundant Lactoferrin and Lactoperoxidase in MAP exposed cattle

Differential expression of two antimicrobial peptides, lactoperoxidase (LPO) and lactoferrin (LF), was also observed in this study. While LPO expression was downregulated in the mandibular (M1, M2) salivary glands, LF expression was decreased in the parotid (P1) salivary gland. In addition to their documented antimicrobial properties and their contribution as innate salivary defense proteins, LF and LPO also function as immunomodulators and serve as regulators of cell growth and differentiation [29, 30]. MAP is an obligate intracellular bacterium that requires mycobactin, an iron-binding siderophore for its growth [31]. Relevantly, via its ability to bind iron, LF deprives microbes from using free iron, which is essential for their survival and thereby exerts an antimicrobial effect [32]. Since the expression of LF was downregulated in MAP exposed animals, this may represent an alternate strategy by MAP to enhance iron uptake; however, this is only speculation until further characterization can be performed. In this study, it is interesting that *LF* and *LPO* expression were reduced in MAP exposed animals. Since they are both secreted and detected in bovine saliva [19], they could offer potential as putative salivary biomarkers to augment MAP diagnosis in cattle.

Another KEGG pathway that was over-represented and was common between mandibular and parotid salivary glands (M1, M2 and P1) was the ‘complement and coagulation cascades pathway’ (KEGGID = bta04610). The DEGs identified in this pathway included complement genes such as: complement *C3* in M1 and M2; complement *C2*, complement factor *B* and complement factor *1* in M1; and complement *C4-A-like* DEG in the P1 salivary gland. Functioning as opsonins, complement proteins and Fcγ receptors enhance uptake of MAP by macrophages and this is believed to be a strategy by which MAP escapes host defenses, by residing and replicating undetected within macrophages [33, 34]. The expression of all the complement DEGs and an Fc γ receptor (FCGR1A) was upregulated in MAP exposed animals in our study implying potential increased MAP intake by host cells. Consistent with this, both complement C3 and Fcγ receptor (FCGR1A) were identified in KEGG pathway ‘phagosome’ (KEGGID = bta04145). In another transcriptomic analysis, increased expression of complement proteins was also reported in mice experimentally infected with MAP [35].

Other DEGs with immunoregulatory properties identified in this study, with previously reported associations with MAP infection, were *TIMP1* (inhibitor of matrix metalloproteinase 1) and *TNFRSF21* (tumor necrosis factor receptor superfamily member 21). These genes were differentially expressed in the M1 salivary glands, with expression of *TIMP1* and *TNFRSF21* being upregulated in MAP exposed cattle. This finding is in agreement with a previous study where the authors reported increased expression of *TIMP1* and *TNFR1* (member of TNF receptor superfamily similar to *TNFRSF21*) in peripheral blood mononuclear cells of cows infected with MAP implicating these genes with reduced tissue remodeling and increased apoptotic activity, respectively, in infected animals [36]. Quantitative trait loci (QTL) regions comprising genes containing the *TNFRSF18* and *TNFRSF4* genes that belong to a similar family as *TNFRSF21* were previously found to be associated with antibody response to MAP infection in cattle [37]. Interestingly, some of the DEGs identified in this study, such as *SERPINA5*, *GPX3*, *IGFBP6*, *APOE*, *VWF*, *S100A4*, *IGFBP3*, *CDH13* and *CPB2*, were also reported as markers of early stage *Mycobacterium tuberculosis* infection in humans [38], suggesting a shared etiology between mycobacterial infections.

The limitations associated with the currently available JD diagnostic tests has hindered JD control across the globe. The aim of this study was to gain insight into potential salivary gland biomarkers as an alternative to diagnose MAP exposure. Also, the ease of sampling saliva from animals makes it an excellent matrix for diagnostic testing. Furthermore, the importance of using salivary biomarkers as diagnostic markers for chronic diseases has been reviewed elsewhere [39]. By performing transcriptomic analysis of salivary glands, we identified differentially expressed immune-related genes in cattle challenged with MAP. As indicated earlier, detection of MAP in saliva by PCR has been reported in dairy cattle [23]. Although we did not perform saliva PCR, histopathology was performed to identify any MAP-specific histological lesions in both salivary glands. As no lesions were observed and MAP challenged cattle were sero-positive, as per JD case definition by Whittington et al. [9], the differential transcriptomic changes identified in this study should be viewed only in the context of MAP exposure. Albeit identification of secretory products in saliva was beyond the scope of this study, a global survey of the bovine salivary proteome identified some of the immune DEGs from our study such as *PIGR, LF, LPO,* and complement *C3* [19] (Additional file 4). Identification of common secretory peptides and DEGs in bovine saliva glands highlights their potential use as salivary biomarkers of MAP exposure - subject to validation in cattle of known infection status. While the impact of MAP infection on gut microbiota in calves has been studied [40], the same is not true for the oral cavity. Oral microbial diversity could have important consequences for susceptibility and pathogenesis of Johne’s disease with fecal-oral route as major mode of transmission of infection. Our knowledge regarding oral immunity remains scant. It can be speculated that the transcriptomic differences detected in seroconverted MAP exposed animals might be due to epigenetic changes that could have long-term consequences for gene expression. And the identified proteins might play a key role in superior innate immune response which when subverted might contribute to a failure to clear chronic infection as observed with other Mycobacterial diseases [41–43].

## Conclusions

Studies designed to observe the host response to MAP infection in cattle have mostly focused on intestinal immune response where MAP gains entry from intestinal mucosa into submucosal gut-associated lymphoid tissues (GALT), such as the ileal Peyer’s patches after its ingestion through fecal-oral route [23]. Considering that fecal-oral route serves as the major mode of transmission of MAP infection in cattle, similar insights into the immune response of the oral mucosa are lacking. This study aimed at profiling transcriptomic changes in major salivary glands of cattle post experimental MAP challenge and identified key immune related genes associated with MAP exposure. Although it is difficult to unambiguously ascribe these differential gene products as contributors to MAP pathogenesis, this work has identified a panel of genes that have not previously been associated with MAP exposure in cattle, and thereby shed new light on the pathology of this potentially zoonotic disease. It is possible that elevated expression of the antimicrobial and immunoregulatory proteins identified herein could contribute to the natural resistance of cattle to mycobacterial infection. Future work will aim to profile the secreted peptides in saliva from infected cattle, and at earlier stages of infection to determine their utility as potential biomarkers of infection status.

## Materials and methods

### Experimental infection model

The experimental infection model of this study is previously described in detail [44]. Briefly, thirty-fivecommercially-sourced male Holstein-Friesian calves between three-to-six-weeks of age that constituted the MAP challenged group were orally inoculated on two consecutive days with 2 × 10^9^ CFU of MAP strain *CIT003*; whereas the control group of 20 calves matched with MAP challenged calves by age, breed and sex received a placebo. Blood, serum and fecal samples were collected at regular intervals to determine MAP infection status. Cell mediated immunity was measured using IFN-ɣ assay (Bovigam®) test and serum MAP-specific antibodies were measured using the commercially available IDEXX ELISA kit. Fecal samples were cultured for 42 days using the TREK ESP para-JEM system (Thermo Scientific).

### Salivary gland excision and preservation

At the end of the experimental infection trial, cattle were euthanized via intra-jugular administration of pentobarbital sodium (Release 300 mg/ml, Chanelle Veterinary, Galway, Ireland or Euthatal 200 mg/ml, Merial Animal Health, Harlow, United Kingdom) and underwent immediate post-mortem examination. Parotid and mandibular salivary glands, the two large cattle salivary glands, were collected from 18 MAP challenged and 6 control cattle. Both the glands were sampled from their respective dorsal (P1 and M1) and ventral extremities (P2 and M2) as shown in Fig. 2a. As both are large glands, sampling was done at dorsal and ventral extremities to verify and compare any transcriptomic differences within each gland. At each anatomical part, sterile and disposable scalpels and forceps were used to prevent any protein cross-contamination. For RNA-Seq transcriptome analysis, samples were immediately stored in liquid nitrogen and transported to the laboratory and stored at − 80 degree Celsius until further use. Cross-sections of each salivary gland tissue extremity were collected and stored in 10% formalin prior to histopathology.

### Histopathology

Formalin fixed salivary gland samples were dehydrated through graded alcohol before being embedded in paraffin wax. Sections of 5 μm thickness were made and then stained with hematoxylin and eosin (H&E) stains for histopathology to detect epithelioid macrophage microgranulomas [45, 46]; and by Ziehl-Nielsen’s (ZN) staining method for detection of acid fast bacteria which stains MAP in red [46].

### RNA extraction, library preparation and RNA-sequencing

Salivary gland tissue samples from control (*n* = 5) and MAP challenged (*n* = 5) cattle were selected for RNA-Seq transcriptome analysis. Animals selected from the MAP challenged group were all sero-positive for MAP-specific antibodies at least once during the experimental period as measured by IDEXX ELISA kit and will be referred to as MAP exposed group [9]. All the control cattle were repeatedly and consistently negative for ELISA and fecal culture test throughout the study. ELISA test and fecal culture results are provided in Additional file 5: Table S5. For each animal, parotid (P1 and P2) and mandibular (M1 and M2) salivary gland samples were homogenized in Trizol, following which RNA was extracted using RNeasy Mini Kit (Qiagen) as per manufacturer’s instructions. RNA quantity and quality were assessed using both a nanodrop spectrophotometer and the Agilent 2100 bioanalyzer. The average RIN value of all the samples was > 7, excluding for M2 sample of animal 2176 in the infected group whose RIN value was very low and was not included in further library preparation and analysis. TruSeq (Illumina TruSeq RNA Library v2 construction) RNA libraries were prepared for all the 39 samples. All libraries were sequenced over Illumina NovaSeq sequencer, generating 100 bp paired end reads (100 million reads/sample).

### Quality control, mapping and differential read count quantification

FASTQC was used to assess the quality of sequence reads. Low quality reads and adapters were trimmed using Trimmomatic software [47]. Trimmed reads were mapped to Bovine Reference Genome Assembly BTA_UMD3.1 (ftp://ftp.ensembl.org/pub/release-94/fasta/bos_taurus/dna/) using STAR RNA-seq aligner [48] and uniquely mapped read counts per gene/ transcript was derived using STAR --quantMode GeneCounts.

### Differential expression analysis

Differentially expressed genes (DEGs) between MAP exposed and control cattle were identified using the DeSeq2 (v 1.20.0) Bioconductor package in R statistical program [49]. Using median of ratios method, DeSeq2 normalizes raw gene count data by correcting for library size and RNA composition. Pair-wise comparison of each gene between MAP exposed and control cattle is based on negative binomial model to obtain fold changes and associated *p*-values. A False Discovery Rate (FDR) of 5% was used to correct for multiple testing. Finally, genes with *p*_*adj*_ *< 0.05* were considered differentially expressed. Prior to differential expression analysis, normalized read counts of samples were used to generate principal component analysis (PCA) plot to determine sample clustering and to identify outliers within each salivary gland.

### Gene ontology and KEGG pathway analysis of DEGs

Gene ontology and biological pathway analysis was performed using the Clusterprofiler Bioconductor package in R statistical program [50].

## Additional files

Additional file 1: **Table S1.** Post mapping statistics of each salivary gland sample. (XLSX 15 kb)

Additional file 2: **Table S2.** Summary of the identified DEGs in all the 4 salivary gland groups. (XLSX 64 kb)

Additional file 3: **Table S3.** List of identified KEGG pathways in each salivary gland group. (XLSX 16 kb)

Additional file 4: **Table S4.** Lists of all the DEGs from this study that were shared in common with the previous global bovine salivary proteome analysis by Ang et al. (DOCX 12 kb)

Additional file 5: **Table S5.** ELISA and fecal culture test results of control and MAP challenged animals. (XLSX 12 kb)

## Data Availability

All data generated or analyzed during this study are included in this published article and its supplementary information files. The raw data on which this publication is based are available at the Gene Expression Omnibus with the GEO accession number GSE124789 (https://www.ncbi.nlm.nih.gov/geo/query/acc.cgi?acc=GSE124789).

## Abbreviations

DEG: Differentially expressed genes
ELISA: Enzyme-linked immunosorbent assay
G0: Gene Ontology
IFN-γ: Interferon-gamma
JD: Johne’s disease
KEGG: Kyoto encyclopedia of genes and genomes
LF: Lactoferrin
LPO: Lactoperoxidase
MAP: *Mycobacterium avium* subsp. *paratuberculosis*
PCR: Polymerase chain reaction
PIGR: Polymeric immumoglobin receptor
QTL: Quantitative trait loci
RIN: RNA integrity number
TIMP1: Tissue inhibitor of metalloproteinases
TNFRSF18: Tumor necrosis factor superfamily, member 18
TNFRSF4: Tumor necrosis factor superfamily, member 18
TNFSF13: Tumor necrosis factor superfamily, member 13
ZN: Ziehl Nielsen
