# Supplementary material for: Characterization of the bovine salivary gland transcriptome associated with Mycobacterium avium subsp. paratuberculosis experimental challenge
Source: BMC Genomics. 2019 Jun 13;20:491. doi: 10.1186/s12864-019-5845-4 (PMC6567491; doi:10.1186/s12864-019-5845-4)
Supplement: Supplementary file 4 — Table S4. Lists of all the DEGs from this study that were shared in common with the previous global bovine salivary proteome analysis by Ang et al. (DOCX 12 kb) [file 12864_2019_5845_MOESM4_ESM.docx]

**M1 salivary gland group:**

- similar to histone cluster 1, H2bd

- Putative helicase MOV-10

- Retinoic acid receptor responder (Tazarotene induced) 1

- Chloride intracellular channel protein 1

- Similar to S100 calcium-binding protein A11 (Fragment)

- Isoform Long of Polymeric immunoglobulin receptor (PIGR)

- Mucin-1

- SERPINA3-5

- keratin 18

- Metalloproteinase inhibitor 2

- Complement C3 (Fragment)

- Apolipoprotein A-I

- Lactoperoxidase

- Transketolase

**M2 salivary gland group:**

- similar to alpha 1 type XVI collagen

- Cysteine-rich secretory protein 3

**In P1 salivary gland group:**

- Lactotransferrin

**In P2 salivary gland group:**

- Cathepsin L1

- Thrombospondin-1
